# Diabesity: an old term, a modern challenge — multi-omics and precision anti-obesity pharmacotherapy in type 2 diabetes

**DOI:** 10.3389/jpps.2026.17560

**Published:** 2026-10-20

**Authors:** Karolina Hoffmann, Weronika Faustyna Pucek, Wiktoria Skrzypek, Filip Kierzkowski, Piotr Ratajczak, Viviana Maggio, Manfredi Rizzo, Mohamed El-Tanani, Syed Arman Rabbani, Bhoomendra Bhongade, Shakta Mani Satyam, Sirajunisa Talath, Imran Rashid Rangraze, Adil Farooq Wali, Walaa Ibraheem, Yahia El-Tanani, Sorina Ispas, Wiesław Bryl, Anna Paczkowska

**Affiliations:** 1 Department and Clinic of Internal Diseases and Metabolic Disorders, Poznan University of Medical Sciences, Poznań, Poland; 2 Department of Pharmacoeconomics and Social Pharmacy, Poznan University of Medical Sciences, Poznań, Poland; 3 Health Care Complex in Ostrów Wielkopolski, Inpatient Treatment, Department of Internal Medicine – Ward B, Ostrów Wielkopolski, Poland; 4 Promise Department, School of Medicine, University of Palermo, Palermo, Italy; 5 Department of Pharmaceutical Chemistry, Ras Al Khaimah Medical and Health Sciences University, Ras Al Khaimah, United Arab Emirates; 6 Department of Clinical Pharmacy and Pharmacology, RAK College of Pharmacy, RAK Medical and Health Sciences University, Ras Al Khaimah, United Arab Emirates; 7 Department of Pharmacology, RAK College of Medical Sciences, RAK Medical and Health Sciences University, Ras Al Khaimah, United Arab Emirates; 8 Department of Internal Medicine, RAK College of Medicine, RAK Medical and Health Sciences University, Ras Al Khaimah, United Arab Emirates; 9 RAK College of Pharmacy, RAK Medical and Health Sciences University, Ras Al Khaimah, United Arab Emirates; 10 Royal Cornwall Hospital, NHS Trust, Treliske, Truro, United Kingdom; 11 Department of Anatomy, Faculty of Medicine, Ovidius University, Constanta, Romania

**Keywords:** type 2 diabetes mellitus, obesity, multi-omics, precision medicine, incretin-based therapies, systems biology, pharmacometabolomics, body composition

## Abstract

Type 2 diabetes mellitus (T2DM) and obesity are closely interconnected but heterogeneous conditions in which treatment response, safety, body weight, glycemic control, and long-term clinical outcomes vary substantially between individuals. This narrative review evaluates emerging anti-obesity pharmacotherapy in patients with coexisting T2DM and obesity and examines the translational role of multi-omics in future precision treatment selection. Distinct from reviews focused primarily on obesity pharmacotherapy or omics-based disease stratification, the present review emphasizes the interface between contemporary drug mechanisms, T2DM-associated metabolic heterogeneity, candidate molecular signatures, and the level of evidence required for clinically actionable prescribing. We summarize evidence for approved and investigational oral incretin therapies, dual and triple receptor agonists, amylin-based strategies, established antidiabetic drugs, and adjunctive approaches targeting body composition. Throughout the review, we distinguish established clinical evidence from mechanistic rationale, candidate biomarkers, exploratory associations, and hypothetical future precision applications. We also separate metabolic efficacy endpoints, such as body weight and HbA1c, from intermediate cardiometabolic risk markers and from cardiovascular or kidney outcomes demonstrated in dedicated outcome trials. At present, no genomic, metabolomic, lipidomic, proteomic, microbiome, or integrated multi-omics signature has been clinically validated to select a specific anti-obesity medication for patients with T2DM and obesity. Major unresolved issues include the need for prospective biomarker validation, direct comparative trials, standardized assays, externally validated and calibrated prediction models, preservation of skeletal muscle health and physical function, long-term safety, treatment durability, affordability, and equitable implementation. Multi-omics should therefore be viewed as an investigational framework for generating and testing precision-treatment hypotheses rather than as a basis for routine prescribing.

## Introduction

Type 2 diabetes mellitus (T2DM) and obesity are closely interconnected conditions that reinforce one another through insulin resistance (IR), chronic low-grade inflammation, adipose tissue dysfunction, ectopic fat accumulation, and broader cardiometabolic dysregulation [1, 2]. The term “diabesity”, commonly attributed to Ethan A. H. Sims and colleagues in the 1970s, was introduced to emphasize the close metabolic relationship between excess adiposity and diabetes-related dysglycemia [1]. In this review, the term is used descriptively to refer to the coexistence of obesity and T2DM and should not be interpreted as a separate disease entity or independent clinical diagnosis. Coexisting T2DM and obesity are associated with a higher burden of cardiovascular (CV), kidney, hepatic, and functional complications, with important consequences for quality of life (QoL), mortality, and healthcare expenditure [3–5]. Their increasing prevalence reflects changes in lifestyle, nutrition, urbanization, population aging, and environmental exposures as well as substantial biological heterogeneity [6, 7]. Excess adiposity, particularly visceral and ectopic fat accumulation, promotes IR, inflammation, adipokine dysregulation, hepatic steatosis, and progressive impairment of glucose homeostasis. Obesity is therefore both a major risk factor for T2DM and an important modifier of its clinical course, complications, and response to pharmacological treatment [6–10].

The global burden of obesity has increased markedly over recent decades. According to the World Health Organization (WHO), approximately 2.5 billion adults were overweight in 2022, including about 890 million adults with obesity, corresponding to 43% and 16% of the adult population, respectively [6]. Large pooled analyses have shown that adult obesity prevalence more than doubled between 1990 and 2022, while obesity among children and adolescents increased even more rapidly [8]. Projections from the World Obesity Federation (WOF) suggest that the number of adults living with obesity may rise to 1.53 billion by 2035 if current trends continue [7]. This trajectory has major clinical and economic implications, with the annual global economic impact of overweight and obesity projected to exceed USD 4 trillion by 2035 [9].

In parallel, diabetes mellitus (DM) has become a major global health challenge. In 2021, approximately 536.6 million adults aged 20–79 years were estimated to be living with DM worldwide, corresponding to a prevalence of 10.5% [11], and this number was projected to increase to 783.2 million by 2045. More recent International Diabetes Federation estimates indicate that 589 million adults are living with DM and that this number may rise to approximately 853 million by 2050 [12]. Because more than 90% of DM cases are T2DM, the increasing burden of T2DM is closely linked to the global expansion of obesity and related cardiometabolic risk factors [12].

The clinical consequences of coexisting T2DM and obesity extend beyond hyperglycemia and excess body weight [13–15]. Both conditions increase the risk of cardiovascular disease (CVD), chronic kidney disease (CKD), metabolic dysfunction-associated steatotic liver disease (MASLD), obstructive sleep apnea (OSA), heart failure (HF), osteoarthritis, and several obesity-related cancers [10, 13–15]. Importantly, patients with T2DM and obesity do not represent a homogeneous population [16]. Individuals differ in the relative contribution of IR, beta-cell dysfunction, visceral adiposity, liver fat accumulation, inflammation, genetic susceptibility, gut microbiome composition, comorbidity burden, and environmental exposures. This heterogeneity contributes to substantial interindividual variability in therapeutic efficacy, tolerability, risk-factor modification, and treatment durability [10, 13–15].

Genome-wide association studies have identified multiple loci linked to T2DM and CVD [17], but genetic signals explain only part of the observed disease heterogeneity. Contemporary treatment therefore increasingly extends beyond glucose lowering to address body weight, cardiometabolic risk factors, liver disease, and, where supported by dedicated outcome trials, CV and kidney outcomes. These outcome domains should be interpreted separately: reductions in body weight or HbA1c and improvements in blood pressure (BP), lipid profile, liver fat, or albuminuria are clinically relevant but should not automatically be considered evidence of CV or kidney event reduction. Although incretin-based therapies have expanded treatment options, selection among available and emerging therapies remains largely empirical, and validated predictive biomarkers are limited [16, 17].

Previous reviews have examined precision medicine in obesity, multi-omics approaches to obesity management, heterogeneity-based precision medicine in T2DM, and the emerging field of pharmacomulti-omics in diabetes [18–21]. The distinctive contribution of the present review is not the introduction of multi-omics as a new concept, but the integration of the rapidly evolving anti-obesity pharmacotherapy pipeline with an explicit translational hierarchy of evidence in the specific context of coexisting T2DM and obesity. We distinguish population-level clinical efficacy from mechanistic rationale, candidate response biomarkers, exploratory associations, and hypothetical future treatment-selection strategies. Particular emphasis is placed on the gap between biomarker discovery and clinically actionable prescribing, including the validation requirements for treatment combinations, sequencing, body-composition outcomes, and organ-specific risk.

Accordingly, the aim of this narrative review is to summarize contemporary and emerging anti-obesity pharmacotherapies and to critically evaluate how, and at what current level of evidence, multi-omics could contribute to precision pharmacotherapy in patients with coexisting T2DM and obesity.

## Methods and scope of the review

This narrative review was developed through a structured search of peer-reviewed literature complemented by searches of clinical trial registries and official regulatory or sponsor sources. PubMed/MEDLINE was used as the primary bibliographic database. ClinicalTrials.gov was searched to identify ongoing, recently completed, and late-stage development programs relevant to obesity pharmacotherapy and T2DM. Official regulatory documents and company communications were consulted when recent information on regulatory status, development programs, or preliminary results was not yet available in peer-reviewed publications. The search covered literature available through August 2026; no predefined lower publication-date limit was applied.

Search concepts included combinations of terms related to T2DM, obesity, diabesity, precision medicine, multi-omics, genomics, epigenomics, transcriptomics, proteomics, metabolomics, lipidomics, microbiome, pharmacometabolomics, anti-obesity medications, glucagon-like peptide-1 (GLP-1) receptor agonists, glucose-dependent insulinotropic polypeptide (GIP), glucagon receptor agonism, dual receptor agonists, triple receptor agonists, amylin, body composition, treatment response, biomarkers, and the names of individual approved or investigational agents discussed in this review. Priority was given to peer-reviewed randomized controlled trials, particularly phase 2 and phase 3 studies, mechanistic studies, consensus or guideline documents, and human studies evaluating omics-based stratification, biomarkers, pharmacological response, or precision medicine in T2DM and obesity. Studies were considered when they provided clinically or mechanistically relevant information on obesity pharmacotherapy, T2DM, obesity-related metabolic complications, treatment-response heterogeneity, multi-omics, or precision-treatment strategies. Publications were excluded when they were outside the scope of the review, duplicated more complete or more recent evidence, or provided insufficient information on the population, intervention, or outcomes. Preclinical evidence was included selectively when required to explain the mechanism of an emerging pharmacological platform.

The pharmacological scope included established therapies used as contextual comparators, recently approved treatments, agents under regulatory review, and investigational compounds in phase 1, 2, or 3 development for obesity, T2DM, or obesity-related metabolic complications. Because this was a narrative review rather than a systematic review or meta-analysis, no formal protocol registration, duplicate study selection, risk-of-bias assessment, or pooled quantitative synthesis was performed. Clinical trial registry records, regulatory documents, and company communications were used primarily to describe current development or regulatory status and preliminary findings not yet available in peer-reviewed form. Such sources are explicitly distinguished from peer-reviewed randomized clinical evidence and are interpreted as provisional until full data are published. This approach improves transparency but does not eliminate the possibility of selection bias inherent to narrative reviews.

## “Diabesity” as a heterogeneous metabolic phenotype

As it was indicated above, T2DM is not a single disease entity but a heterogeneous syndrome that may arise through different combinations of IR, beta-cell dysfunction, adipose tissue dysfunction, hepatic steatosis, inflammation, altered incretin signaling, gut microbiome changes, and neurobehavioral drivers of food intake. Data-driven clustering studies have identified subgroups of adult-onset DM with differing risks of complications and potentially different therapeutic needs [16]. Genetic and multi-trait analyses further support the presence of mechanistically distinct pathways leading to T2DM, including insulin secretion, insulin action, adiposity distribution, lipid metabolism, and hepatic metabolic traits [22].

In clinical practice, obesity-associated T2DM commonly coexists with visceral adiposity, hepatic IR, MASLD, dyslipidemia, arterial hypertension, OSA, and increased CV risk. Two patients with the same BMI and HbA1c may nevertheless differ substantially in beta-cell reserve, liver fat, inflammatory activity, appetite biology, skeletal muscle status, kidney function, CV vulnerability, and treatment barriers. These differences are clinically relevant, but most have not been validated as predictive biomarkers for selecting one anti-obesity pharmacotherapy over another [16, 17, 22].

Accordingly, current treatment selection should continue to integrate established clinical factors, including glycemic status, obesity severity, cardiorenal and hepatic comorbidities, risk of hypoglycemia, gastrointestinal (GI) tolerability, frailty, nutritional status, patient preference, and access. Molecular or multi-omics profiles may eventually refine this process, but phenotype-treatment associations discussed in this review should be interpreted as hypothesis-generating unless they have been prospectively validated for differential treatment response.

## Multi-omics approaches to precision pharmacotherapy in T2DM

Recent recommendations emphasize management of the cardiometabolic continuum beyond glucose-centered care, including body weight, comorbidities, and long-term patient-centered outcomes [3]. In parallel, the pharmacological landscape of T2DM and obesity has expanded rapidly. When interpreting this evidence, metabolic efficacy endpoints such as weight reduction and HbA1c should be distinguished from intermediate cardiometabolic risk markers and from CV or kidney event reduction demonstrated in dedicated outcome trials.

Multi-omics refers to the integrated analysis of multiple biological layers, including the genome, epigenome, transcriptome, proteome, metabolome, lipidome, and microbiome [23]. In T2DM, these technologies may help move from broad disease labels toward molecularly informed sub-phenotypes and treatment-response signatures [24–26]. Their relevance is particularly strong in obesity-associated T2DM, where metabolic dysfunction involves several interacting organs and pathways rather than isolated hyperglycemia [6, 10, 13, 14, 27].

Genomics can identify inherited variation associated with insulin secretion, adiposity distribution, hepatic lipid metabolism, drug metabolism, and susceptibility to adverse events. Although most common genetic variants individually have modest predictive value, pathway-level genetic information may improve subtype classification and reveal mechanisms that influence drug response [28]. For example, genetic evidence has identified clusters of T2DM risk loci linked to beta-cell dysfunction, proinsulin processing, obesity, lipodystrophy-like fat distribution, and liver-lipid traits [22].

Epigenomics may capture the influence of obesity, hyperglycemia, aging, inflammation, and environmental exposures on metabolic tissues [25, 29]. DNA methylation and chromatin accessibility patterns could eventually help explain metabolic memory, differential reversibility of IR, or long-term response to therapy for weight reduction [30]. Transcriptomics and proteomics may provide functional information on inflammatory pathways, adipose tissue remodeling, hepatic stress, beta-cell stress, endothelial dysfunction, and muscle metabolism [25, 29].

Metabolomics and lipidomics can provide direct information on small-molecule and lipid signatures that reflect the current metabolic state [31, 32]. Branched-chain amino acids, acylcarnitines, bile acids, triglyceride-rich lipoprotein patterns, ceramides, sphingolipids, ketone-related metabolites, and markers of hepatic lipid flux have been associated with IR, lipotoxicity, and MASLD-related phenotypes [33]. Treatment-related changes in these profiles may provide mechanistic or pharmacodynamic information; however, they should not be interpreted as validated pretreatment predictors of response or safety unless prospectively confirmed.

The gut microbiome represents another relevant omics layer because incretin secretion, bile acid metabolism, intestinal barrier function, immune activation, and energy harvest may be influenced by microbial ecology [34]. Although microbiome-guided prescribing is not yet ready for routine T2DM care, microbiome signatures may help explain interindividual variability in GI tolerability, appetite response, and metabolic improvement during incretin-based therapy [35, 36].

The ultimate value of multi-omics will depend on integration and validation rather than on the number of measured molecular layers. Artificial intelligence (AI) and multimodal computational models may support integration of clinical, imaging, digital, and multi-omics data, but their clinical utility depends on interpretability, reproducibility, calibration, external validation, standardized assays, and equitable implementation [37–39]. Throughout this review, three levels of evidence are distinguished: established clinical evidence from randomized trials, regulatory studies, or routine practice; mechanistic or biomarker evidence that may explain heterogeneity but does not constitute a prescribing criterion; and proposed future multi-omics-guided treatment selection, which remains investigational. At present, no validated multi-omics algorithm can reliably select among individual anti-obesity pharmacotherapies in patients with T2DM and obesity.

## Systems biology of the gut-brain-liver-adipose-muscle axis

Energy balance and glucose homeostasis are regulated through a network linking the gut, central nervous system (CNS), liver, adipose tissue, skeletal muscle, pancreas, kidney, and CV system. Gut-derived hormones such as GLP-1, GIP, glucagon, peptide YY, and amylin modulate appetite, gastric emptying, insulin secretion, glucagon secretion, hepatic metabolism, and satiety signaling [40–46]. GLP-1 receptor activation reduces appetite, slows gastric emptying, improves glucose-dependent insulin secretion, and lowers glucagon secretion. GIP receptor signaling influences insulin secretion and adipose tissue biology, and its pharmacological role appears context-dependent. Glucagon receptor agonism may increase hepatic energy expenditure and lipid oxidation, which is relevant to MASLD/MASH phenotypes but may also require careful monitoring of heart rate (HR), tolerability, and glycemic effects [47–49]. Amylin contributes to meal termination, satiety, and potentially reward-related eating behavior [45, 46].

From a systems-biology perspective, emerging therapies modulate inter-organ pathways rather than acting solely through appetite suppression. Multi-omics profiling may help map gut-brain-liver-adipose tissue-skeletal muscle communication by identifying tissue-specific and circulating signatures of response. For example, high liver fat, inflammatory activity, or a lipotoxic metabolomic profile provides a mechanistic rationale for investigating whether therapies with substantial weight and hepatic effects produce differential benefit in particular subgroups. Similarly, sarcopenic obesity provides a rationale for prioritizing preservation of skeletal muscle health and resistance exercise. These examples are hypothesis-generating and have not been validated as treatment-selection criteria; prospective biomarker-enriched trials are required to test treatment-by-biomarker interactions.

## Established and emerging incretin-based and multi-receptor therapies in T2DM with obesity

### Liraglutide as an established GLP-1 receptor agonist and reference therapy

Liraglutide is an established GLP-1 receptor agonist that provides an important reference point for understanding the evolution of incretin-based pharmacotherapy in T2DM and obesity. In the SCALE Obesity and Prediabetes trial, once-daily subcutaneous liraglutide 3.0 mg, combined with lifestyle intervention, produced clinically significant weight reduction and improved metabolic control in adults with overweight or obesity without T2DM [50]. In adults with overweight or obesity and T2DM, the SCALE Diabetes trial showed that liraglutide 3.0 mg resulted in greater weight reduction than placebo [51], while the SCALE Insulin trial confirmed its efficacy in patients with T2DM and overweight or obesity treated with basal insulin [52]. At the lower antidiabetic dose, liraglutide 1.8 mg also demonstrated CV benefit in the LEADER trial, in which it reduced major adverse cardiovascular events (MACE) in patients with T2DM and high CV risk [53]. In addition, longer-term data from the SCALE Obesity and Prediabetes trial showed that liraglutide 3.0 mg reduced the risk of progression to T2DM in individuals with prediabetes and obesity or overweight [54]. Although the magnitude of weight reduction achieved with liraglutide is generally lower than that observed with newer agents such as semaglutide, tirzepatide, and investigational multi-receptor agonists, liraglutide remains clinically important because it established GLP-1 receptor activation as a therapeutic strategy capable of improving glycemic control, body weight, and cardiometabolic outcomes. In the context of precision pharmacotherapy for T2DM with obesity, liraglutide also illustrates the need to understand interindividual variability in response to GLP-1 receptor agonism and to identify biomarkers that may guide treatment selection.

### Oral incretin therapies

The therapeutic landscape includes approved therapies, agents under regulatory review, and investigational compounds at different stages of development. Because this landscape changes rapidly, each therapy is identified below according to its regulatory or clinical-development status as of August 2026. Evidence from approved therapies, phase 3 programs, phase 2 trials, and early-phase studies should not be considered equivalent. Likewise, population-level efficacy and safety data should be distinguished from proposed precision-medicine applications, because molecular or multi-omics predictors have generally not been prospectively validated for treatment selection.

As of August 2026, oral semaglutide 25 mg had received FDA approval for chronic weight management. Peer-reviewed phase 3 evidence demonstrated clinically meaningful weight reduction in adults with overweight or obesity, while regulatory status was additionally documented by the manufacturer and regulatory sources [55, 56]. The oral formulation provides a non-injectable alternative to subcutaneous semaglutide, although administration requirements and GI tolerability may influence adherence. Weight reduction and glycemic effects should be interpreted separately from CV or kidney outcomes; organ-level benefit should only be inferred where supported by dedicated outcome evidence.

Orforglipron, a non-peptide oral GLP-1 receptor agonist, demonstrated weight reduction in peer-reviewed phase 2 and phase 3 trials before Food and Drug Administration approval in April 2026 [57–59]. The approval status is based on regulatory or manufacturer information, whereas efficacy estimates derive from the corresponding clinical trials. As a small-molecule agent, orforglipron can be administered without the fasting and water-intake restrictions required for oral peptide semaglutide.

Together, these agents provide clinical evidence that non-injectable incretin therapy is feasible for chronic weight management. Their use should be guided by approved indications, efficacy, tolerability, contraindications, patient preference, cost, and adherence considerations rather than by unvalidated molecular profiles.

### GLP-1/GIP dual agonism

Tirzepatide is an approved dual GIP/GLP-1 receptor agonist with established weight and glycemic efficacy [60]. VK2735 and enicepatide (CT-388) remain investigational. VK2735 is in phase 2/3 development, with injectable and oral formulations under evaluation [61–63]. Enicepatide has progressed through early and phase 2 clinical development [64–66]. Preliminary publicly reported mid-stage results suggested substantial weight reduction, but non-peer-reviewed results should be regarded as provisional until complete trial data are published. Future precision-medicine studies should evaluate whether IR, beta-cell reserve, appetite burden, or metabolomic signatures modify response to dual agonism; these features are candidate response modifiers rather than validated treatment-selection biomarkers.

The clinically relevant question is therefore not whether emerging dual agonists can be assigned to molecular phenotypes today, but whether prospective comparative studies can demonstrate reproducible treatment-by-biomarker interactions. Direct comparator trials and biomarker-enriched studies are required before such profiles can be used for differential prescribing.

### GLP-1/GIP/glucagon triple agonism

Retatrutide is an investigational phase 3 triple receptor agonist targeting GLP-1, GIP, and glucagon receptors. In a peer-reviewed phase 2 trial, retatrutide produced dose-dependent weight reduction approaching 24% at 48 weeks, with additional reductions in liver fat reported in a MASLD study [67, 68]. These findings provide clinical and mechanistic evidence but do not establish a biomarker-defined subgroup for preferential use. More recently, sponsor-reported topline results from the phase 3 TRIUMPH-1 trial indicated a mean weight reduction of 28.3% at 80 weeks with the 12-mg dose, and an extension subgroup reportedly reached 30.3% at 104 weeks [69, 70]. Because these phase 3 results were available primarily through sponsor communication at the time of manuscript preparation, they should be considered preliminary until full peer-reviewed data are available. The ongoing TRIUMPH-Outcomes trial is specifically evaluating CV and kidney outcomes [71]; until those data are available, weight reduction and changes in metabolic risk markers should not be interpreted as evidence of CV or kidney protection.

### GLP-1/glucagon dual agonism

Survodutide, pemvidutide, and mazdutide combine GLP-1 receptor agonism with glucagon receptor activation [47–49]. Survodutide has peer-reviewed phase 2 evidence in obesity and MASH and has progressed to phase 3 obesity development [47, 48]. Its hepatic effects provide a mechanistic rationale for investigation in metabolic liver disease, but liver fat or metabolic profiles are not validated biomarkers for preferential treatment selection. Long-term CV safety and dedicated clinical outcomes require separate evaluation.

Pemvidutide has peer-reviewed phase 2 data showing effects on body weight and hepatic outcomes in MASLD/MASH populations [49, 72]. These findings support further clinical development but should not be interpreted as proof that a hepatic molecular or lipidomic profile can select pemvidutide over other therapies. Phase 3 comparative and outcome data are required to establish durable efficacy, tolerability, and any organ-specific clinical benefit [47, 48, 73].

Mazdutide has phase 3 randomized clinical data in Chinese adults with overweight or obesity, with additional development in populations that include T2DM [74–76]. Reported effects include weight reduction and improvements in several metabolic and hepatic risk markers. These surrogate changes are clinically relevant but should remain distinct from demonstrated CV or kidney outcome benefits. GI adverse events, particularly nausea, vomiting, and diarrhea, remain important tolerability considerations [76].

### Amylin-based and GLP-1/amylin therapies

CagriSema, amycretin, petrelintide, cagrilintide, and eloralintide incorporate amylin biology into obesity treatment [77–83]. Persistent hunger, large meal size, or appetite dysregulation provide a biological rationale for studying amylin-based strategies, but these clinical features have not been validated as predictive biomarkers of differential response. Current treatment selection should therefore be based on clinical evidence, regulatory status, safety, and patient factors rather than on presumed hyperphagic phenotypes.

#### CagriSema

CagriSema combines cagrilintide, a long-acting amylin analogue, with semaglutide. Phase 3 trials have demonstrated substantial weight reduction, including in patients with T2DM, with additional effects on HbA1c, waist circumference (WC), and BP [77, 78, 84]. As of August 2026, CagriSema remained under FDA regulatory review for weight management following submission of a new drug application [85, 86]. GI adverse events remain the principal tolerability concern. The complementary mechanisms of GLP-1 and amylin provide a rationale for studying appetite-related response modifiers, but no such biomarker has been prospectively validated for treatment selection.

Head-to-head evidence should be interpreted separately from indirect or network comparisons. CagriSema’s ultimate clinical position will depend on regulatory decisions, comparative effectiveness, tolerability, and the populations studied. Sponsor-reported head-to-head results available in 2026 should be regarded as preliminary until fully peer-reviewed [87].

Network meta-analysis suggests that several high-efficacy incretin and amylin-based strategies can produce large average reductions in body weight, with GI adverse events increasing as treatment intensity rises [88]. Such analyses are useful for contextualization but do not substitute for direct randomized comparisons and should not be used to infer biomarker-defined superiority for individual patients.

#### Amycretin

Amycretin is an investigational unimolecular GLP-1/amylin receptor agonist with oral and subcutaneous development programs [79, 80]. Early clinical data support continued evaluation of dose-dependent weight reduction, but the evidence remains early phase. Its dual mechanism provides a biological rationale for studying appetite and satiety phenotypes; however, longer randomized trials are required to define efficacy, tolerability, body-composition effects, and whether any pretreatment characteristic predicts differential benefit.

#### Petrelintide

Petrelintide is an investigational long-acting amylin analogue in phase 2 development [81]. Potential roles as monotherapy, an adjunct to incretin therapy, or part of maintenance strategies remain under investigation. Appetite-related phenotypes are biologically plausible research targets but should not be treated as validated indications for preferential selection. Longer studies are needed to determine efficacy, tolerability, durability, and functional body-composition outcomes.

#### Cagrilintide

Cagrilintide is a long-acting amylin analogue developed for once-weekly administration. A phase 2 dose-finding trial demonstrated dose-dependent weight reduction, with GI adverse events as the main tolerability issue [82]. Clinical studies of co-administration with semaglutide support the feasibility of combination therapy [89], and pharmacokinetic data suggest that renal or hepatic impairment does not substantially alter exposure or tolerability [90]. These findings support continued evaluation of cagrilintide in combination strategies; they do not establish an appetite phenotype or omics signature that should currently guide selection.

#### Eloralintide

Eloralintide (LY3841136) is an investigational selective, long-acting amylin receptor agonist administered once weekly. Phase 1 proof-of-concept and phase 2 data indicate clinically relevant weight reduction with a tolerability profile that requires continued evaluation [83, 91, 92]. The available evidence supports further development of this non-incretin mechanism, but its role as monotherapy or in combination with incretin-based agents and any predictive biomarker profile remain unvalidated.

### Other anti-obesity medications under investigation

Several additional agents broaden the pharmacological landscape beyond conventional incretin agonism. Maridebart cafraglutide (MariTide) is an investigational once-monthly conjugate combining GLP-1 receptor agonism with GIP receptor antagonism and has phase 2 clinical data with phase 3 development underway [93–95]. CT-996 (RO7795081) is an investigational oral small-molecule GLP-1 receptor agonist in early and dose-ranging clinical development in overweight/obesity and T2DM [96, 97]. Development-stage evidence should be interpreted according to trial phase and publication status rather than grouped with approved therapies.

Setmelanotide is an FDA-approved melanocortin-4 R agonist for specific genetically defined or syndromic obesity indications and is not a treatment for common polygenic obesity [98]. Bimagrumab, an investigational activin type II R pathway blocker, represents an approach targeting body composition rather than appetite alone. In a phase 2 clinical trial in adults with T2DM and obesity, bimagrumab reduced fat mass and increased lean mass [99]. These therapies illustrate the importance of matching claims to the population and indication actually studied.

Collectively, these agents illustrate the diversity of contemporary obesity pharmacotherapy, including oral, less frequently administered, genetically targeted, and body-composition-oriented approaches. Their place in precision treatment will depend on regulatory status, comparative effectiveness, safety, population-specific evidence, and prospective validation of any proposed biomarkers.

Table 1 summarizes the regulatory and clinical-development status, studied populations, principal efficacy and safety findings, evidence maturity, and potential biomarker relevance of selected therapies. Table 2 provides omics domains and their current translational maturity.

**TABLE 1:** Selected approved and investigational anti-obesity pharmacotherapies discussed in this review (regulatory/development status as of August 2026).

Agent	Status/key population	Mechanism; route/frequency	Weight and glycemic effects (references cited in this review)	Major AEs/body composition (references cited in this review)	Evidence source/maturity	Potential biomarker or phenotype relevance
Tirzepatide	FDA-approved; obesity/overweight and T2DM populations	GIP/GLP-1 RA; SC weekly	Substantial weight reduction; strong HbA1c lowering in T2DM [60]	GI AEs; body-composition data available, including lean-mass change [100]	Peer-reviewed phase 3 RCTs; established clinical use	No validated omics selector; conventional clinical indications guide use
Oral semaglutide 25 mg	FDA-approved for weight management; adults with overweight/obesity	GLP-1 RA; oral daily	Clinically meaningful weight reduction; glycemic effects consistent with GLP-1 pharmacology [55, 56]	Mainly GI AEs; body-composition evidence limited for this formulation	Peer-reviewed phase 3 RCT + regulatory/manufacturer status	No validated omics selector
Orforglipron	FDA-approved April 2026; obesity programs, with T2DM development	Small-molecule GLP-1 RA; oral daily	Dose-dependent weight reduction; glycemic benefit in T2DM development [57–59]	GI AEs; body-composition data limited	Peer-reviewed phase 2/3 RCTs + regulatory/manufacturer status	No validated omics selector
CagriSema	Under FDA regulatory review; obesity/overweight with and without T2DM	Cagrilintide + semaglutide; SC weekly	20.4% mean weight reduction at week 68 in REDEFINE 1 using the treatment-policy estimand; 22.7% with the trial-product estimand; lower magnitude in T2DM, with HbA1c reduction reported in T2D programs [77, 78, 84–86]	GI AEs; detailed skeletal-muscle outcomes not yet established	Peer-reviewed RCT data plus registry/sponsor regulatory information	Appetite-related phenotypes are hypothetical; no validated selector
Retatrutide	Phase 3; primarily obesity/overweight; MASLD and cardiorenal-risk programs	GLP-1/GIP/glucagon triple RA; SC weekly	Up to ≈24% at 48 weeks in peer-reviewed phase 2; 28.3% at 80 weeks sponsor-reported phase 3 topline [67–71]	GI AEs; long-term body-composition and safety data incomplete	Peer-reviewed phase 2 + registry + sponsor-reported phase 3 topline	Hepatic/metabolic profiles are research hypotheses only
Survodutide	Phase 3 obesity development; obesity and MASH populations	GLP-1/glucagon dual RA; SC weekly	Clinically meaningful weight reduction; hepatic effects in MASH studies [47, 48]	GI AEs; HR and hepatic safety require monitoring	Peer-reviewed phase 2 RCTs + phase 3 registry	MASLD phenotype is mechanistically relevant but not validated for selection
Pemvidutide	Phase 2 completed; phase 3 obesity program; MASLD/MASH populations	GLP-1/glucagon dual RA; SC weekly	Weight reduction with liver-fat and hepatic-marker improvements [49, 72, 73]	GI AEs; body-composition evidence limited	Peer-reviewed phase 2 + development/registry information	Hepatic/lipidomic profiles remain hypothetical selectors
Mazdutide	Phase 3 clinical program; Chinese overweight/obesity populations including T2DM subgroups	GLP-1/glucagon dual RA; SC weekly	Substantial weight reduction with glycemic and metabolic-marker improvement [74–76]	Nausea, vomiting, diarrhea; body-composition data limited	Peer-reviewed phase 3 RCT evidence in studied populations	No validated molecular selector; generalizability requires confirmation
Amycretin	Early phase/phase 2 development; overweight/obesity	Unimolecular GLP-1/amylin RA; oral or SC	Early dose-dependent weight reduction; definitive glycemic effects not established [79, 80]	GI tolerability under evaluation; body composition not established	Early-phase clinical evidence	Appetite/satiety signatures are hypothetical
Petrelintide	Phase 2; overweight/obesity with weight-related comorbidity	Long-acting amylin analogue; SC weekly	Weight reduction under phase 2 evaluation; glycemic effects not established [81]	Tolerability and functional body composition under evaluation	Phase 2 clinical development	Hyperphagic phenotype is hypothetical
Cagrilintide	Phase 2 monotherapy/combination evidence	Long-acting amylin analogue; SC weekly	Dose-dependent weight reduction; combination with semaglutide supports greater reduction than semaglutide alone in early studies [82, 89]	GI AEs; detailed functional body-composition endpoints limited	Peer-reviewed phase 2/phase 1b clinical evidence	No validated response biomarker
Eloralintide	Phase 2; overweight/obesity with comorbidity	Selective long-acting amylin RA; SC weekly	Clinically relevant weight reduction in phase 1/2 studies; glycemic effects not established as primary outcome [83, 91, 92]	GI tolerability appears manageable in early studies; body-composition evidence incomplete	Peer-reviewed phase 1/2 evidence	No validated response biomarker
VK2735	Phase 2/3; obesity/overweight; T2DM-specific development planned/ongoing	GIP/GLP-1 RA; SC weekly and oral formulation in development	Dose-dependent weight reduction in phase 2; glycemic evidence less mature than approved dual agonists [61–63]	GI AEs; body-composition data limited	Peer-reviewed phase 2 + phase 3 registry	No validated omics selector
Enicepatide (CT-388)	Phase 2 with later-phase development announced; obesity with/without T2DM	GIP/GLP-1 RA; SC weekly	Substantial preliminary weight reduction; complete peer-reviewed mid-stage efficacy data limited [64–66]	GI AEs; body-composition data limited	Early peer-reviewed data + non-peer-reviewed development reporting	No validated omics selector
MariTide	Phase 3; obesity with/without T2DM	GLP-1 agonism + GIP receptor antagonism; SC monthly	Phase 2 weight reduction demonstrated; glycemic effects evaluated in T2DM subgroup/programs [93–95]	GI AEs; body-composition data limited	Peer-reviewed phase 2 + phase 3 development	No validated omics selector
CT-996/RO7795081	Phase 1/2; overweight/obesity and T2DM	Small-molecule GLP-1 RA; oral daily	Early efficacy and glycemic data; definitive magnitude not yet established [96, 97]	Early safety/tolerability evaluation; body composition not established	Early-phase trial/registry evidence	No validated omics selector
Setmelanotide	FDA-approved for specific monogenic/syndromic obesity; not common polygenic obesity	MC4 receptor agonist; SC daily	Weight reduction demonstrated in genetically defined populations; not a general T2DM-obesity therapy [98]	Hyperpigmentation, injection-site and GI reactions; body composition not primary focus	Dedicated phase 3 + FDA-approved genetic indication	Genotype/diagnosis is an established indication-defining biomarker
Bimagrumab	Investigational phase 2; adults with T2DM and obesity	Activin type II receptor pathway blockade; IV	Reduced fat mass with modest body-weight change; metabolic improvement [99]	Increased lean mass; muscle-oriented safety/functional outcomes require further study	Peer-reviewed phase 2 RCT	Potential relevance to sarcopenic obesity remains investigational

Legend: AEs, adverse events; FDA, US, food and drug administration; GIP, glucose-dependent insulinotropic polypeptide; GLP-1, RA, glucagon-like peptide-1, receptor agonist; GI, gastrointestinal; MASLD/MASH, metabolic dysfunction-associated steatotic liver disease/steatohepatitis; MC4, melanocortin-4; RA, receptor agonist; SC, subcutaneous; T2DM, type 2 diabetes mellitus. Evidence categories are descriptive and do not represent formal GRADE, ratings. Sponsor-reported topline results and registry information are not considered equivalent to peer-reviewed randomized clinical evidence.

**TABLE 2:** Omics domains, representative biomarker classes, therapeutic relevance, and current translational maturity in precision pharmacotherapy of T2DM with obesity.

Omics domain	Representative biomarkers/signatures	Potentially linked mechanism/class	Potential application	Evidence maturity (references cited in this review)	Validated for treatment selection?
Genomics	SLC2A2, GLP1R and other pathway variants	Metformin; GLP-1 RA and related pathways	Candidate prediction of pharmacological response	Candidate; some human pharmacogenetic associations [21, 22, 28, 101]	No, except genetic diagnosis for specific monogenic therapies such as setmelanotide
Epigenomics	DNA methylation and chromatin-accessibility signatures	Multiple metabolic pathways	Characterization of metabolic memory and tissue state	Exploratory/mechanistic [23, 25, 29, 30]	No
Transcriptomics	Adipose, liver, skeletal-muscle, immune-cell and gut expression profiles	Multiple classes	Mapping active pathways during response/nonresponse	Exploratory/mechanistic [23, 25, 29]	No
Proteomics	Inflammatory, vascular, hepatic, kidney and endocrine proteins	Multiple classes	Candidate efficacy, safety, or organ-risk markers	Exploratory/candidate [23, 25, 29]	No
Metabolomics/lipidomics	BCAA-related metabolites, acylcarnitines, bile acids, ceramides, sphingolipids, triglyceride-rich species	Metformin, sulfonylureas, incretin-based therapies and other metabolic agents	Candidate baseline response signatures; pharmacodynamic monitoring	Candidate or exploratory; several human associations [24, 26, 31–33, 102, 103]	No
Microbiome	Microbial composition, bile-acid metabolism, intestinal-barrier and immune signatures	Incretin-related and metabolic pathways	Hypothetical prediction of tolerability or metabolic response	Exploratory/hypothetical [34–36]	No
Integrated multi-omics + AI	Combined molecular, clinical, imaging, digital and behavioral data	Cross-class decision support	Future patient stratification and treatment selection	Investigational; requires calibration, external validation, assay standardization and prospective utility studies [38, 39, 104]	No

Legend: AI, artificial intelligence; BCAA, branched-chain amino acid; GIP, glucose-dependent insulinotropic polypeptide; GLP-1, RA, glucagon-like peptide-1, receptor agonist; T2DM, type 2 diabetes mellitus. “Candidate” indicates human treatment-response evidence without prospective validation for clinical selection; “exploratory” indicates an association requiring replication; “hypothetical” indicates biologically plausible but currently insufficient clinical evidence.

### Developmental uncertainty and the need for clinical differentiation

The expanding anti-obesity drug pipeline should be interpreted cautiously. Biological plausibility, receptor novelty, or short-term metabolic effects do not necessarily translate into durable clinical efficacy, acceptable tolerability, or practical long-term use. Danuglipron, for example, demonstrated dose-dependent weight reduction in a phase 2b trial but illustrates how tolerability, dosing practicality, and safety can affect development decisions [105, 106]. Similarly, proof-of-concept efficacy should not be equated with regulatory success or superiority over established therapy.

### Body composition and functional outcomes

Substantial weight reduction may involve losses of both fat mass and lean mass; however, lean mass should not be considered synonymous with skeletal muscle mass [100, 107–113]. Depending on the assessment method, lean mass includes skeletal muscle as well as other non-fat tissues and body water. Therefore, changes in DXA-derived lean mass do not directly quantify loss of contractile skeletal muscle. More muscle-specific assessment may include appendicular lean mass and muscle cross-sectional area or volume assessed by computed tomography (CT) or magnetic resonance imaging (MRI). Muscle health should also be evaluated functionally: handgrip strength, chair-stand performance, gait speed, the Short Physical Performance Battery, and other validated measures provide information that cannot be inferred from body composition alone. This distinction is particularly important in older adults and in patients with frailty or sarcopenic obesity. A treatment-associated reduction in lean mass should not automatically be interpreted as clinically meaningful skeletal muscle deterioration unless supported by evidence of reduced skeletal muscle quantity, strength, or physical performance. Future trials should therefore combine body-composition and functional endpoints rather than relying on body weight or lean mass alone.

## Omics-informed biomarkers of therapeutic response and safety in patients with T2DM and obesity

For clarity, biomarkers in this review are classified according to clinical maturity. Established clinical biomarkers or variables are routinely used in care, although none currently constitutes an omics-based tool for selecting a specific anti-obesity pharmacotherapy. Candidate biomarkers are molecular or metabolic features supported by human treatment-response data but not prospectively validated for treatment selection. Exploratory associations are signals identified mainly in cross-sectional, *post hoc*, or mechanistic analyses that require independent replication. Hypothetical future biomarkers are biologically plausible markers proposed from systems biology or pathway-level reasoning but currently lack sufficient clinical evidence. At present, no genomic, metabolomic, lipidomic, proteomic, microbiome, or integrated multi-omics signature is validated for selecting among GLP-1 receptor agonists, dual or triple receptor agonists, amylin-based therapies, or other emerging anti-obesity medications (AOM) in patients with T2DM and obesity.

Established clinical variables that inform treatment decisions include BMI, waist circumference (WC), duration of T2DM, HbA1c, beta-cell reserve, kidney function, CVD, MASLD status, eating behavior, medication history, and previous intolerance. Genomic, epigenomic, transcriptomic, proteomic, metabolomic, lipidomic, and microbiome data may add biological resolution, but the available evidence is heterogeneous. Metabolomic and lipidomic signatures related to IR, lipotoxicity, hepatic dysfunction, or branched-chain amino acid metabolism are best regarded as candidate or exploratory markers unless prospective treatment-by-biomarker interactions have been demonstrated [22, 24–33, 102]. Microbiome signatures associated with bile-acid metabolism, incretin secretion, GI tolerability, or metabolic response remain exploratory or hypothetical and are not validated prescribing tools [34–36].

Pharmacometabolomics illustrates the distinction between association and clinical utility. Cross-sectional studies by Naja and colleagues identified metabolite patterns associated with good or poor response to metformin and sulfonylureas [24, 26], but these signatures have not been prospectively validated for choosing therapy. Pharmacogenetic evidence is similarly promising but incomplete. For example, SLC2A2 rs8192675 has been associated with differential HbA1c response to metformin [21], and GLP1R rs10305420 has been associated with glycemic response to liraglutide in one clinical population [101]. These findings are candidate biomarkers rather than established prescribing criteria.

Predictive biomarkers should also be distinguished from pharmacodynamic biomarkers. A predictive biomarker should identify, before treatment initiation, differential benefit from one therapy compared with another. By contrast, a pharmacodynamic marker reflects a biological change after treatment exposure and may clarify mechanism without supporting pretreatment drug selection. Exploratory metabolomic and lipidomic changes during tirzepatide therapy, including changes in branched-chain amino acid-related metabolites and lipid species, are examples of pharmacodynamic signatures rather than validated pretreatment predictors [103]. Similarly, preserved C-peptide secretion may predict some clinical responses to GLP-1 receptor agonists in insulin-treated T2DM, but the available evidence remains limited and does not constitute an omics-based selector [114]. Table 3 summarizes representative examples and their validation status.

**TABLE 3:** Representative biomarkers and molecular signatures associated with pharmacological response in T2DM and obesity and their current validation status.

Biomarker/signature	Drug/class	Study population	Endpoint/reported quantitative result (references cited in this review)	Biomarker maturity	Validation for treatment selection
SLC2A2 rs8192675	Metformin	MetGen consortium; 13,123 participants of different ancestries; primary analysis 10,577 of european ancestry	C Allele associated with 0.17 percentage-point greater HbA1c reduction; among participants with obesity, CC vs. TT associated with 0.33 percentage-point greater reduction [21]	Candidate pharmacogenetic biomarker	No routine clinical validation; requires prospective utility evidence
Untargeted metabolomic signature including sphingomyelins, acylcholines and glutathione-related metabolites	Metformin	Adults with T2DM receiving metformin	Good vs. poor glycemic response; multivariate metabolomic discrimination reported in cross-sectional study [26]	Exploratory pharmacometabolomic association	No prospective external validation
Metabolomic signature including acylcholines, gamma-glutamyl amino acids, sphingomyelins and related metabolites	Sulfonylureas	Adults with T2DM receiving sulfonylurea therapy	Good vs. poor glycemic response; multivariate discrimination reported in cross-sectional analysis [24]	Exploratory pharmacometabolomic association	No prospective validation
GLP1R rs10305420	Liraglutide/GLP-1 RA	233 iranian adults with T2DM receiving liraglutide	TT genotype associated with optimal glycemic response: OR 3.28, 95% CI 1.41–7.65; P = 0.006 [101]	Candidate pharmacogenetic biomarker	No independent prospective prescribing validation
Random C-peptide>=0.8 ng/mL	GLP-1 RAs in insulin-treated T2DM	Retrospective cohort of insulin-treated adults with T2DM	Insulin discontinued in 11/23 with C-peptide>=0.8 ng/mL vs. 0/7 with lower C-peptide; P = 0.029 [114]	Candidate clinical predictor; not an omics marker	Not prospectively validated as a comparative treatment selector
BCAA/BCKA and triglyceride/diglyceride metabolomic-lipidomic changes	Tirzepatide/GIP-glp-1 RA	259 adults with T2DM from phase 2b randomized trial	Treatment-induced changes correlated with improvements in HbA1c, HOMA2-IR and proinsulin; no pretreatment predictive effect established [103]	Exploratory pharmacodynamic signature	No; reflects on-treatment biology rather than validated pretreatment selection

Quantitative results are presented using the effect measure or performance metric reported in the original study and should not be directly compared across rows because study designs and statistical methods differ. None of the omics-based biomarkers in this table is currently validated for routine selection of anti-obesity pharmacotherapy in patients with T2DM, and obesity. A treatment-induced molecular change is pharmacodynamic and should not be interpreted as a pretreatment predictive biomarker.

## Established drugs, repurposing, and rational combination therapy in T2DM with obesity

Combination and sequencing strategies differ substantially in evidentiary maturity. Some combinations are supported by clinical or real-world evidence, whereas phenotype-directed or omics-directed combinations remain hypothesis-generating. This distinction is important because a combination may be clinically reasonable on the basis of established cardiorenal or glycemic indications even when no biomarker-guided selection strategy has been validated.

Metformin remains foundational therapy in many treatment pathways. Sodium-glucose cotransporter 2 (SGLT2) inhibitors have demonstrated CV and kidney benefits in appropriate T2DM populations and may be selected according to established CKD, HF, or CV indications [115–122]. These dedicated outcome data should be distinguished from surrogate improvements in body weight, HbA1c, BP, lipids, or albuminuria. Thiazolidinediones may remain useful in selected insulin-resistant or MASLD-predominant patients because of effects on insulin sensitivity and hepatic steatosis, although weight gain, edema, and HF risk limit broader use [123, 124]. Incretin therapy combined with basal insulin and cagrilintide combined with semaglutide are additional examples supported by randomized clinical data [89, 125].

By contrast, preferential use of pioglitazone according to a multi-omics IR signature, glucagon-containing therapies according to a hepatic lipidomic profile, or amylin-based therapy according to a hyperphagic molecular phenotype remains investigational. These strategies are mechanistically plausible but require prospective comparative studies showing treatment-by-biomarker interactions.

Similarly, induction-maintenance strategies, treatment de-escalation, and sequencing after substantial weight reduction require dedicated trials. Future precision algorithms should only be implemented if they improve patient-relevant outcomes beyond high-quality conventional selection based on glycemic status, CV and kidney indications, hepatic disease, adverse events risk, functional status, patient preference, and access.

## Controversies and unresolved issues in precision pharmacotherapy of T2DM with obesity

The pharmacological landscape of T2DM with obesity has changed rapidly, with therapies producing effects across several distinct domains. These domains should not be conflated: body weight reduction and glycemic improvement are metabolic efficacy endpoints; BP, lipid profile, liver fat, inflammation, and albuminuria are intermediate or surrogate measures; and CV death, MACE, HF events, CKD progression, and kidney failure are clinical outcomes requiring dedicated outcome evidence.

### Is greater weight loss always better?

The field increasingly values large reductions in body weight, but the optimal balance among efficacy, tolerability, nutritional adequacy, glycemic control, CV benefit, preservation of skeletal muscle quantity, and maintenance of muscle strength and physical performance remains uncertain. In older adults and in patients with frailty, sarcopenic obesity, chronic illness, or advanced diabetic complications, functional outcomes may be more clinically consequential than an isolated reduction in DXA-derived lean mass [100, 107–113].

### Durability and treatment discontinuation

Weight regain after discontinuation of incretin-based therapy indicates that long-term treatment may be required for many patients [126, 127]. In the STEP 1 extension, participants regained a substantial proportion of previously lost weight after withdrawal of semaglutide 2.4 mg [126]. Withdrawal of tirzepatide in SURMOUNT-4 was similarly associated with weight regain and partial reversal of several cardiometabolic risk-marker improvements [127]. Whether multi-omics can identify patients with durable remission or high relapse risk remains hypothetical and requires prospective validation.

### Need for direct comparative and biomarker-enriched trials

Most development programs remain placebo-controlled rather than directly comparative. Head-to-head trials among established and emerging therapies are needed to determine comparative efficacy, tolerability, body-composition effects, and clinically relevant outcomes. Biomarker-enriched trials should prespecify candidate markers and formally test treatment-by-biomarker interactions; retrospective associations alone cannot establish predictive utility.

### Safety, tolerability, and clinical differentiation

GI adverse events, gallbladder disease, pancreatitis concerns, heart-rate changes, dehydration, malnutrition, and changes in skeletal muscle quantity or function require appropriate long-term evaluation [47, 48, 67, 108–113, 128, 129]. For investigational multi-receptor therapies, improvements in weight, HbA1c, BP, lipids, or liver fat should not be described as CV or kidney protection unless confirmed in dedicated outcome trials. The same evidentiary distinction applies to emerging safety signals: early tolerability data are not equivalent to long-term safety characterization.

### Equity and implementation

Multi-omics technologies and high-cost pharmacotherapies may widen disparities if implementation is limited to selected populations. Practical translation therefore requires affordable and standardized assays, interoperable data systems, transparent algorithms, population diversity, and reimbursement models that do not restrict access to clinically indicated therapy. Equity should be evaluated prospectively rather than assumed from model performance in selected research cohorts.

## Discussion

The current evidence supports a clear distinction between population-level therapeutic efficacy and precision treatment selection. Randomized clinical trials have established that several incretin-based and emerging anti-obesity therapies can produce substantial average improvements in body weight and glycemic control, and selected established drug classes have dedicated CV or kidney outcome evidence. However, these findings do not establish that genomic, metabolomic, lipidomic, proteomic, or microbiome profiles can currently select one anti-obesity therapy over another in an individual patient.

This distinction is central to the interpretation of the proposed framework. Biological features such as hepatic steatosis, IR, appetite dysregulation, inflammation, or sarcopenic obesity may provide a mechanistic rationale for studying differential response to glucagon-containing, amylin-based, incretin-based, or body-composition-oriented strategies. Mechanistic plausibility, treatment-induced molecular changes, and retrospective biomarker associations are nevertheless insufficient to establish predictive utility. A further distinction is required between metabolic efficacy and organ-specific clinical benefit: improvements in body weight, HbA1c, BP, lipid profile, liver fat, or albuminuria may indicate favorable risk modification but cannot be assumed to reduce CV or kidney events without dedicated outcome evidence.

Predictive biomarkers must also be separated from pharmacodynamic signatures. Many omics studies identify molecular changes during treatment or correlate baseline features with response, but such findings do not necessarily predict, before treatment, which therapy will provide greater benefit than an alternative. Clinical precision prescribing requires prespecified treatment-by-biomarker interactions, replication in independent and diverse cohorts, standardized and reproducible assays, calibration and external validation of prediction models, and demonstration of incremental value beyond conventional clinical variables.

The principal contribution of this review is therefore not the introduction of a new omics technology or disease classification, but the integration of contemporary anti-obesity pharmacology with an explicit translational hierarchy of evidence. By separating established population-level effects from candidate molecular predictors, exploratory pharmacodynamic signatures, and hypothetical phenotype-treatment associations, the framework clarifies where precision pharmacotherapy currently stands and what evidence is still required before multi-omics can influence routine prescribing in T2DM with obesity.

Clinical translation will require more than statistically significant associations. Multi-omics models must resist overfitting, remain calibrated and transportable across populations and platforms, and be supported by standardized assays and transparent reporting. Most importantly, prospective studies must show that algorithm-guided decisions improve patient-relevant outcomes, safety, persistence, or efficiency compared with high-quality conventional care. Until these requirements are met, multi-omics should be considered a research framework for generating and testing precision-treatment hypotheses rather than an established component of routine prescribing. The major challenge is no longer simply to identify additional molecular associations, but to determine which signals are predictive, reproducible, clinically actionable, and sufficiently informative to change treatment decisions beyond established clinical assessment.

## Current research gaps and future directions for precision pharmacotherapy in T2DM with obesity

First, future clinical trials should move beyond average treatment effects and incorporate prespecified clinical, metabolic, imaging, and multi-omics phenotyping. Candidate biomarkers should be defined before analysis and tested for treatment-by-biomarker interactions rather than merely correlated with outcome. Replication in independent cohorts is necessary to determine whether a marker is truly predictive of differential treatment response rather than simply prognostic or mechanistically plausible.

Second, trials should deliberately include clinically relevant T2DM subgroups, including early and long-duration disease, MASLD/MASH, CKD, HF, older adults, sarcopenic obesity, and participants from diverse ancestry and socioeconomic groups. External validity requires that predictive signatures be reproducible across populations, laboratories, platforms, and healthcare settings [17, 47, 130–135].

Third, future studies should prespecify distinct outcome domains. Metabolic efficacy should include body weight, HbA1c, time in range, insulin or sulfonylurea de-intensification, and beta-cell function. Intermediate organ or risk markers may include liver fat, MASH resolution, BP, lipids, or albuminuria. CV and kidney protection should be reserved for adjudicated clinical outcome evidence from appropriately designed trials. Body-composition endpoints should distinguish total lean mass from skeletal muscle quantity and should be complemented by muscle strength and physical performance, including validated measures such as handgrip strength, chair-stand performance, gait speed, or the Short Physical Performance Battery [100, 109, 110, 113].

Fourth, AI and computational modeling may facilitate integration of high-dimensional clinical, imaging, digital, and multi-omics data, but methodological rigor is essential before implementation. Because the number of molecular features may greatly exceed the number of participants, multi-omics models are particularly vulnerable to overfitting. Development should therefore include appropriate internal validation followed by independent external validation in temporally, geographically, and demographically distinct cohorts. Discrimination should be accompanied by calibration assessment, because accurate separation of responders from non-responders does not necessarily imply accurate estimation of individual treatment benefit. Reproducibility may also be compromised by batch effects, sample handling, storage conditions, platform differences, reagent lots, preprocessing, normalization, and analytical pipelines. Harmonized pre-analytical procedures, assay standardization, quality-control protocols, and transparent reporting are prerequisites for transportability [23, 38, 39, 104, 136, 137].

Fifth, statistical validity alone is insufficient to establish clinical utility. Candidate algorithms should be tested prospectively to determine whether biomarker- or multi-omics-guided treatment selection improves efficacy, safety, treatment persistence, patient-reported outcomes, or resource use compared with high-quality conventional assessment. Combination and sequencing strategies likewise require dedicated randomized trials; clinically supported combinations should be distinguished from phenotype-directed hypotheses that remain unvalidated.

Finally, pediatric and adolescent populations remain an important evidence gap. Trials should evaluate not only weight and glycemic outcomes but also growth, puberty, bone health, nutritional adequacy, psychological outcomes, body composition, muscle function, and durability after treatment discontinuation. Precision approaches in younger populations should not be extrapolated directly from adult omics or pharmacotherapy data.

## Conclusion

Coexisting obesity and T2DM represent a heterogeneous metabolic phenotype requiring treatment strategies that extend beyond glycemic control and body weight reduction alone (Figure 1). Emerging incretin-based, amylin-based, multi-receptor, and oral therapies, together with approaches targeting body composition, have broadened the therapeutic landscape. Their selection, however, remains guided primarily by conventional clinical characteristics, approved indications, comorbidities, safety, and patient preferences rather than by validated molecular profiles.

**FIGURE 1:**
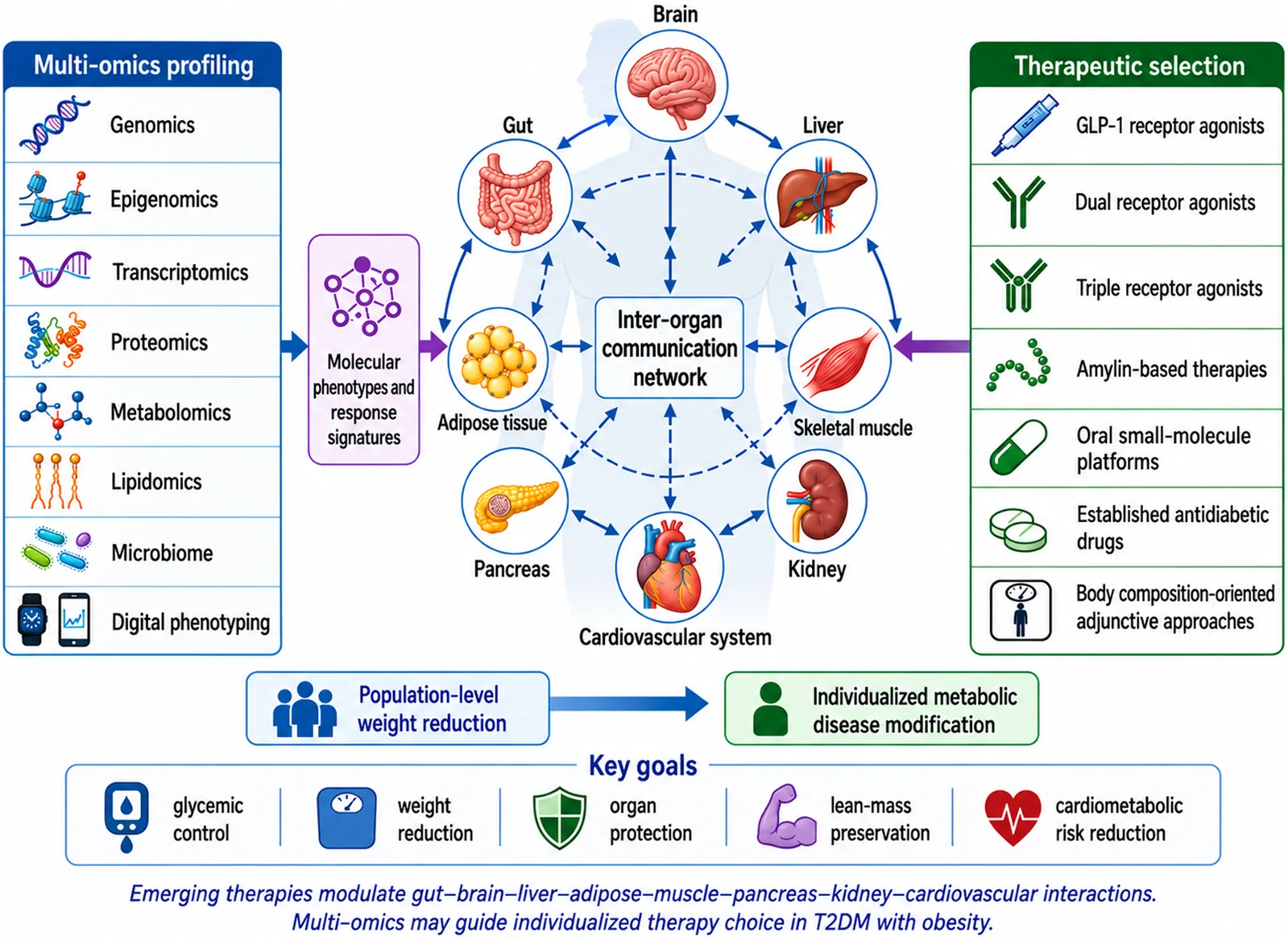
Systems biology framework of precision anti-obesity pharmacotherapy in T2DM. The figure illustrates a conceptual systems biology framework for precision anti-obesity pharmacotherapy in T2DM with obesity. Multi-omics profiling, including genomics, epigenomics, transcriptomics, proteomics, metabolomics, lipidomics, microbiome analysis, and digital phenotyping, may support the identification of molecular phenotypes and response signatures. These signatures may help guide therapeutic selection among GLP-1 RAs, dual and triple receptor agonists, amylin-based therapies, oral small-molecule platforms, established antidiabetic drugs, and body composition-oriented adjunctive approaches. The central network highlights bidirectional communication among the gut, brain, liver, adipose tissue, skeletal muscle, pancreas, kidney, and cardiovascular system, emphasizing that emerging therapies may act as modulators of inter-organ metabolic regulation rather than isolated weight-loss agents. The proposed framework aims to shift treatment from population-level weight reduction toward individualized metabolic disease modification, with key goals including glycemic control, weight reduction, organ protection, lean-mass preservation, and cardiometabolic risk reduction. Abbreviations: GLP-1 RAs, glucagon-like peptide-1 receptor agonists; T2DM, type 2 diabetes mellitus.

Multi-omics may ultimately refine patient stratification and treatment selection, but its current role remains investigational. Translation into routine precision pharmacotherapy will require prospectively validated predictive biomarkers, standardized assays, externally validated and calibrated algorithms, direct comparative evidence, and demonstration that biomarker-guided decisions improve clinically relevant outcomes beyond standard care.

## Data Availability

The original contributions presented in this review are included in the article. No original datasets were generated or analyzed. All data discussed are derived from publicly available sources cited in the reference list. Further inquiries can be directed to the corresponding author.

## Glossary

AI: artificial intelligence
BMI: body mass index
BP: blood pressure
CEA: cost-effectiveness analysis
CKD: chronic kidney disease
CNS: central nervous system
CRP: C-reactive protein
CT: computed tomography
CV: cardiovascular
CVD: cardiovascular disease
DM: diabetes mellitus
DPP-4: dipeptidyl peptidase-4
FDA: Food and Drug Administration
FGF21: fibroblast growth factor 21
GDF15: growth differentiation factor 15
GI: gastrointestinal
GIP: glucose-dependent insulinotropic polypeptide
GLP-1 RA: glucagon-like peptide-1 receptor agonist
HbA1c: glycated hemoglobin
HR: heart rate
IR: insulin resistance
IV: intravenous
MACE: major adverse cardiovascular events
MASH: metabolic dysfunction-associated steatohepatitis
MASLD: metabolic dysfunction-associated steatotic liver disease
MRI: magnetic resonance imaging
N/A: not available
OSA: obstructive sleep apnea
PYY: peptide YY
QoL: quality of life
RWE: real-world evidence
SGLT2: sodium-glucose cotransporter 2
SC: subcutaneous
T2DM: type 2 diabetes mellitus
TZD: thiazolidinedione
WC: waist circumference
WHO: World Health Organization
WOF: World Obesity Federation
